# Temporal stability of human heading perception

**DOI:** 10.1167/jov.23.2.8

**Published:** 2023-02-14

**Authors:** Mufaddal Ali, Eli Decker, Oliver W. Layton

**Affiliations:** 1 Department of Computer Science, Colby College, Waterville, ME, USA

**Keywords:** heading, self-motion, temporal dynamics, optic flow, neural model

## Abstract

Humans are capable of accurately judging their heading from optic flow during straight forward self-motion. Despite the global coherence in the optic flow field, however, visual clutter and other naturalistic conditions create constant flux on the eye. This presents a problem that must be overcome to accurately perceive heading from optic flow—the visual system must maintain sensitivity to optic flow variations that correspond with actual changes in self-motion and disregard those that do not. One solution could involve integrating optic flow over time to stabilize heading signals while suppressing transient fluctuations. Stability, however, may come at the cost of sluggishness. Here, we investigate the stability of human heading perception when subjects judge their heading after the simulated direction of self-motion changes. We found that the initial heading exerted an attractive influence on judgments of the final heading. Consistent with an evolving heading representation, bias toward the initial heading increased with the size of the heading change and as the viewing duration of the optic flow consistent with the final heading decreased. Introducing periods of sensory dropout (blackouts) later in the trial increased bias whereas an earlier one did not. Simulations of a neural model, the Competitive Dynamics Model, demonstrates that a mechanism that produces an evolving heading signal through recurrent competitive interactions largely captures the human data. Our findings characterize how the visual system balances stability in heading perception with sensitivity to change and support the hypothesis that heading perception evolves over time.

## Introduction

Self-motion creates a streaming pattern of motion on the eye known as optic flow that contains rich information about the movement of the observer through the world (Gibson, 1950; Warren, 1995, 1998; Fajen, 2021). A prominent example is the global pattern of motion that radiates outward from a single point that is experienced by an observer moving straight through the environment without eye movements. This point is known as the focus of expansion (FoE) and specifies the observer’s direction of movement (heading) (Gibson, 1950). Decades of research have demonstrated that humans are indeed capable of accurately judging their heading direction from optic flow, to within 1${}^{\circ }$ under idealistic conditions (Warren, Morris, & Kalish, 1988; Van Den Berg, 1992). Although the optic flow field may contain coherent global structure, local portions undergo tremendous change from one moment to the next. To appreciate this, consider the visual experience of walking through a dense forest along a straight path. Despite the consistent global outflow from the FoE, each passing tree produces fast motion that occupies an increasing portion of the optic flow field until it suddenly exits the field of view. Remarkably, this frequent, tremendous flux on the retina does not challenge human heading perception—it remains stable and robust.

To achieve stability in heading perception, the visual system faces a challenging problem: it must maintain sensitivity to the sensory flux that accompanies actual changes in self-motion while disregarding fluctuations that do not. An over-reliance on sensory signals could result in erratic, constantly shifting heading estimates, even when the observer’s heading does not actually change (Layton & Fajen, 2016a). In contrast, suppressing too much sensory flux could give rise to sluggish hysteresis, where the heading estimate persists long after heading has changed. How the visual system balances these factors to subserve stable heading perception is not well-understood.

One solution could involve the accumulation of sensory evidence over time. Heuer and Britten (2004) report evidence for such a process within the dorsal medial superior temporal area (MSTd), which is involved in heading perception (Britten & Van Wezel, 2002; Gu, Deangelis, & Angelaki, 2012). In the study, monkeys indicated which of two optic flow patterns was present within a variable coherence random dot display (i.e., a proportion of the dots moved in random directions). Thresholds with which the responses of MSTd neurons could be used to discriminate between the preferred optic flow pattern from the “opposite” pattern decreased over time. While thresholds started to plateau once the optic flow viewing duration reached approximately 400-ms, they improved over the subsequent 600-ms of viewing by an additional approximately 25%. It is important to emphasize that these findings were obtained from an optic flow pattern discrimination task, so the extent to which they translate to heading perception is unclear. Nonetheless, these findings show that integrating optic flow over time in MSTd may facilitate optic flow selectivity when faced with sensory uncertainty. Neural dynamics that resemble evidence accumulation processes have been documented elsewhere in the primate brain, such as in areas MT (Britten, Shadlen, Newsome, & Movshon, 1992; Cook & Maunsell, 2002), LIP (Roitman & Shadlen, 2002; Gold & Shadlen, 2007), and M1 (Thura & Cisek, 2016).

Computational models may offer insight into how an evidence accumulation process when implemented in biologically plausible neural mechanisms could promote stability in heading estimation. However, many biologically inspired neural models of heading perception base their heading estimates exclusively on a single vector field representation of the optic flow field and therefore do not address the dynamics of heading perception over time (Perrone, 1992; Lappe & Rauschecker, 1993; Perrone & Stone, 1994; Royden 1997, 2002; Beintema & Van Den Berg, 1998; Warren & Saunders, 1995; Beyeler et al., 2016). The Competitive Dynamics (CD) model of heading estimation in the primate dorsal stream on the other hand offers an account wherein each model MSTd neuron accumulates evidence about the preferred heading direction over time from optic flow signals (Browning, Grossberg, & Mingolla, 2009b; Layton, Mingolla & Browning, 2012; Layton & Fajen, 2016a, 2017; Steinmetz, Layton, Powell, & Fajen, 2022). Heading-sensitive neurons within the CD model interact with each other using recurrent on-center/off-surround connections. This increases the contrast over time between the signals generated by neurons that collect the strongest and weakest evidence for their preferred headings—the activations of “winning” neurons are enhanced, whereas those of other neurons decrease. Recurrent computations have long been used in computer vision algorithms, such as the Kalman filter (Wu, Rink, Caelli, & Gourishankar, 1989; Barron & Eagleson, 1996), to integrate new sensory evidence with existing self-motion estimates. The CD model is a dynamical system, so multiple neurons may survive the competition and contribute toward the heading estimate, for example, by “voting” for their preferred heading direction in proportion to their respective activations. Owing to the recurrent signals, heading estimates reflect the recent time history, not simply the instantaneous heading. To influence the heading estimate, the input signal corresponding with a particular heading must be sufficiently strong over a long enough period of time to overcome the inhibitory signals sent by the present winning neurons in the network. As a dynamical system, the requirements for such an influence to occur change over time and depend on the relative strength between winning neurons in the network and the input optic flow signal, the degree of compatibility of the signals over the recent time history, and the parameters of the network. In general, strong optic flow inputs should overtake existing winners in the network more quickly than weak, noisy inputs. This means that the network is capable of suppressing or dampening the effect of brief fluctuations in the optic flow, which supports the stability of heading estimation.

Simulations of the CD model indicate that recurrent interactions among heading tuned neurons may play an important role in stabilizing heading perception, especially in dynamic environments wherein the observer moves in the presence of an independently moving object (Layton & Fajen, 2016a). Such moving objects complicate heading perception because the optic flow within the contours of the object is not directly compatible with the observer’s heading through the environment. The presence of moving objects may decrease the accuracy of human heading judgments, but, remarkably, by only several degrees even though the object may occupy much of the visual field (Warren & Saunders, 1995; Royden & Hildreth, 1996; Layton & Fajen 2016c, 2016b, 2017). Simulations show that the CD model produces stable human-like estimates over time, while estimates from models that do not perform temporal integration may diverge and change erratically (Layton & Fajen, 2016a). The recurrent competition allows signals corresponding with the true heading to persist temporarily without substantial influence from the locally conflicting signals that arise from the moving object.

**Figure 1.**
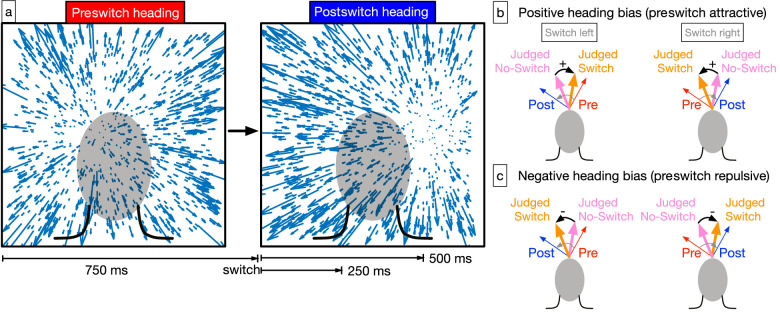
Overview of the stimulus paradigm and heading bias convention. (a) On switch trials subjects viewed optic flow consistent with an initial heading direction (“preswitch heading”). After 750-ms, the heading direction switched to a different heading direction (“postswitch heading”). The postswitch optic flow appeared for either 250-ms or 500-ms. No-switch trials consisted only of optic flow corresponding to the postswitch period. (b, c) If the preswitch optic flow affects perception of the postswitch heading (blue), it may bias heading judgments (orange) toward the preswitch heading (red). We define heading bias as the difference in heading judgments on switch (orange) and no-switch (pink) trials. Positive heading bias indicates bias toward the preswitch heading (b; attractive effect) and negative heading bias indicates bias away from preswitch heading (c; repulsive effect). Cases illustrating left (left column) and right (right column) switches are shown. Angles are exaggerated for visual clarity.

In the present study, we investigated the stability of human heading perception from optic flow when the direction of self-motion changes. As Figure 1a illustrates, the observer experiences simulated self-motion along a particular direction (“preswitch heading”) followed by a shift in heading toward a new direction (“postswitch heading”). We examined the extent to which the preswitch heading influences perception of the postswitch heading. Based on the sensory integration mechanism in the CD model, we would expect bias in judgments of the postswitch heading toward the preswitch heading. This is because it takes time in the model for the sensory signal corresponding with the postswitch heading to overwhelm the evolving signal that is based on the preswitch heading.

There are at least two different ways in which the preswitch heading could influence judgments of the postswitch heading. One possibility is that the preswitch heading exerts an attractive effect, which means that the judged final heading is biased toward the initial heading relative to judgments of the final heading when the trial did not contain a switch (Figure 1b). The comparison with the judged final heading without a switch is important because it accounts for heading error unrelated to the switch. The CD model would predict an attractive influence because optic flow signals corresponding with the postswitch heading would combine with evolving heading signals and shift the estimate toward the postswitch heading over time as evidence accumulates. Another possibility is that the preswitch heading exerts a repulsive effect on judgments, which means that the judged final heading is biased away from the initial heading relative to judgments of the final heading when the trial did not contain a switch (Figure 1c). Neural adaptation, the reduction in neural sensitivity owing to the prolonged exposure to a stimulus, represents one mechanism by which repulsion may occur (Kohn, 2007; Webster, 2015). Repulsive effects have been observed in the tuning of neurons in primary visual cortex (Saul & Cynader 1989b, 1989a; Dragoi, Sharma, & Sur, 2000).

## Experiment 1: Temporal stability of heading perception

The aim of Experiment 1 was to investigate whether the heading before a change in self-motion direction (preswitch heading) influences human heading perception after the switch (postswitch heading). In the experiment, subjects judged their heading after viewing simulated self-motion. On trials with a heading switch (“switch condition”), optic flow consistent with the preswitch heading appeared for 750-ms, followed by a sudden switch in optic flow consistent with the postswitch heading. We selected the 750-ms preswitch duration to encourage a stable heading percept (Layton & Fajen, 2016c). We independently controlled the size of the heading shift (“switch angle”) between the preswitch and postswitch headings based on the prediction from the CD model that larger switch angles should increase the influence of the preswitch heading, because it should take longer for evidence about the more dissimilar optic flow field to overwhelm the evolving estimate.

We independently varied the duration of the postswitch optic flow—250-ms or 500-ms. The CD model predicts that longer viewing time should improve the accuracy of heading judgments given that it provides more time for evidence to accumulate about the postswitch heading.

To dissociate the influence of eccentricity on the judgment of the postswitch heading (Sun, Zhang, Alais, & Li, 2020; Cuturi & Macneilage, 2013) from that of the heading switch, we included control trials that consisted of simulated self-motion along only one of the possible postswitch headings, but without the switch (“no-switch condition”). We subtracted heading judgments obtained in no-switch trials from those obtained in switch trials that have the same final postswitch heading. We refer to this bias mediated by the switch as “heading bias” (Figure 1b, c). We interleaved the switch and no-switch trials randomly within each block so that subjects remained vigilant and did not develop expectations about the presence of a switch.

### Methods

#### Participants

Twelve college students between the age of 18 and 21 years participated in the study for monetary compensation. All were naïve with respect to the purpose of the experiment, and had normal or corrected-to-normal vision. We obtained written consent from all participants before the experiment started. The experimental protocol was approved by the Institutional Review Board at Colby College and is in compliance with the Declaration of Helsinki.

#### Visual stimuli

Subjects viewed displays (65${}^{\circ }$ W × 40${}^{\circ }$ H) showing simulated translation in the horizontal plane through the center of a rigid three-dimensional (3D) volume (depth: 1.5–9 m) filled with 1,650 white spherical particles (diameter in the virtual environment: 3.5 cm) rendered on a black screen using the Unity game engine (Movies S1 and S2). The virtual observer translated at 1.5 m/s. The stimuli were generated on a Dell workstation equipped with a NVIDIA GeForce RTX 2080 Ti graphics card at the frame rate of 60 Hz and displayed on a 32-inch Dell Ultrasharp 4K U3219Q monitor.

We define heading as the azimuthal angle with respect to the cardinal depth (“Z”) axis in the world that is aligned with the straight-ahead of the virtual observer. Simulated self-motion consists of pure translation within the present study (i.e., the view of observer does not rotate), so the heading angle is the same in both world-relative and observer-relative reference frames. Trials that contained a switch in heading direction (switch condition) started with simulated self-motion along headings of 0${}^{\circ }$, $\pm 6^{\circ }$, where 0${}^{\circ }$ refers to the straight-ahead direction (i.e., toward center of screen), positive heading angles refer to right-of-straight-ahead headings, and negative heading angles refer to left-of-straight-ahead headings. We randomly jittered the preswitch heading on each trial by at most $\pm 1^{\circ }$.

The postswitch heading is the sum of the preswitch heading and the switch angle. We selected switch angles of $\pm 3^{\circ }$, $\pm 6^{\circ }$, and $\pm 12^{\circ }$, where negative angles refer to leftward shifts in heading and positive angles refer to rightward shifts. For example, a −6${}^{\circ }$ preswitch heading combined with a 12${}^{\circ }$ switch angle results in a 6${}^{\circ }$ postswitch heading. We selected these preswitch heading and switch angles to confine postswitch headings to the central 36${}^{\circ }$ [−18${}^{\circ }$, 18${}^{\circ }$] of the visual field because the accuracy of human heading perception degrades in the periphery (Sun et al., 2020; Cuturi & Macneilage, 2013).

#### Procedure

Subjects initiated each trial by clicking a mouse button. Each switch trial consisted of 750-ms of simulated translation along the preswitch heading, a switch in heading, and 250-ms or 500-ms of simulated translation along the postswitch heading. No-switch trials consisted of simulated translation along one of the heading directions used in the postswitch phase of switch trials. The duration was either 250-ms or 500-ms, matching the length of the postswitch phase of switch trials. Subjects were instructed to fixate and maintain gaze on a central fixation cross throughout the experiment. The last frame of the stimulus froze on-screen at the end of the trial and a thin yellow probe (V: 4.5${}^{\circ }$) appeared at a random horizontal position along the vertical midline of the display. Subjects were instructed to adjust the probe position by moving the mouse and align it with the perceived heading direction. After confirming the selection with a mouse click, subjects pressed the spacebar key to initiate the next trial.

Each subject completed 360 trials blocked by repetition (5) of the experimental conditions in a fully crossed design. Each block consisted of 36 trials with a switch (3 preswitch heading angles × 6 switch angles × 2 postswitch durations) and 36 trials without a switch (headings matched postswitch headings from corresponding switch trials). The experiment lasted approximately 60 minutes.

At the beginning of the experiment, subjects completed a short practice heading judgment task without switches in heading to become familiar with the experimental instructions. No feedback was provided in the practice task or main experiment.

#### Analysis

To quantify the accuracy of human heading judgments, we first computed the heading error E on each trial as

(1) $$E=H^{\text{judged}}-H^{\text{true}},$$

where $H^{\text{judged}}$ refers to the judged heading and $H^{\text{true}}$ refers to the true heading, which represents the postswitch heading angle on switch trials or the heading angle on no-switch trials.

Statistical analyses involved heading bias, a measure that quantifies the influence of the preswitch heading on heading judgments of the postswitch heading:

(2) $$B_{s,p}=E_{s,p}^{\text{switch}}-{\bar{E}}_{s,p}^{\text{no}}$$

In Equation 2, $B_{s,p}$ defines the heading bias for subject s and postswitch heading angle p, $E_{s,p}^{\text{switch}}$ defines the heading error for the same subject and postswitch heading angle, and ${\bar{E}}_{s,p}^{\text{no}}$ defines the mean heading error on no-switch trials for the same subject and postswitch heading angle. We omit subscripts for other independent variables that do not constrain the computation for simplicity—switch angle, postswitch duration, and preswitch heading angle.

We enforced the following sign convention when plotting data to facilitate the interpretation of the results around the main hypothesis: positive heading bias means a bias toward the preswitch heading (attractive effect) and negative heading bias means bias away from the preswitch heading (repulsive effect). This was achieved by negating bias values associated with positive switch angles computed according to Equation 2. Figure 1d,c schematizes the bias after enforcing this convention.

When we used repeated measures analysis of variance (ANOVA) in statistical analyses of the bias data, we verified sphericity using Mauchly’s test. In cases where sphericity did not hold, we report Greenhouse-Geisser corrected degrees of freedom and *p* values.

#### CD model simulations

The CD model is a biologically inspired, dynamical neural model of primate brain areas MT and MSTd that estimates both heading and object motion during self-motion (Layton & Fajen, 2016a; Layton & Niehorster, 2019; Layton & Fajen, 2020) and builds on the ViSTARS model (Browning, Grossberg, & Mingolla, 2009a, 2009b). We simulated a simplified version of the model focused on heading estimation that accounts for human heading perception in the presence of independently moving objects (Layton, Mingolla, & Browning, 2012). Other versions of the CD model include that of Layton and Fajen (2016a), which estimates heading based on the motion detected from a sequence of images; that of Steinmetz et al. (2022), which implements dynamic tuning that adapts to recently viewed optic flow properties; and that of Layton and Niehorster (2019) and Layton and Fajen (2020), which focus on the recovery of object motion in a world-relative reference frame. Compared with the model of Layton and Fajen (2016a), the Layton et al. (2012) version omits the many dynamically interacting neural processing layers that correspond with primate brain areas LGN, V1, MT, and MSTd. This difference stems from the simplifying assumption that optic flow is not estimated from a sequence of images (i.e., vector field representations of the optic flow serve as the model input). Given that the displays in the present study consist of simulated self-motion through a static environment, the substantial increase in complexity of the Layton and Fajen (2016a) does not seem necessary. The versions of Layton and Niehorster (2019) and Layton and Fajen (2020) contain a number of components that are not relevant here, such as disparity tuning and object motion estimation. We selected the model of Layton et al. (2012) for simulations in the present study to focus on the contributions of the recurrent neural network in MSTd that accumulates sensory evidence about heading, a mechanism that all versions of the CD model have in common.

We briefly summarize the structure of the model of Layton et al. (2012) in the remainder of this section and provide parameter values used in present simulations in Table 1. Please refer to Layton et al. (2012) for model equations and more detail. The model code is available on GitHub: https://github.com/owlayton/NM-Competitive-Dynamics-Simple_Release.

**Table 1.** Parameters used in CD model simulations. Variable naming follows conventions used in Layton et al. (2012).

Parameter	Description	Value
	Number of frames per optic flow sample	34 frames
	Optic flow spatial resolution	128 × 128 pixels ($90\times 90^{\circ }$)
$r_{MT}$	MT RF radius	6 pixels (4.2${}^{\circ }$)
$\sigma_{MT}$	MT spatial pooling filter standard deviation	9.51 pixels (6.7${}^{\circ }$)
$r_{MST}$	MSTd cross-heading template match pooling filter radius	19 pixels (13.4${}^{\circ }$)
σMST	MSTd cross-heading template match pooling filter standard deviation	10.08 pixels (7.1${}^{\circ }$)
A	MSTd cell passive decay rate	0.81
B	MSTd cell excitatory potential	146
c	Exponential temporal moving average constant	0.7

We simulated the model with sequences of analytically defined optic flow that resemble the conditions of Experiment 1. We generated optic flow consistent with virtual observer (field of view: 90${}^{\circ }$) translating through a 3D dot cloud of 10,000 dots. We simulated of 18 distinct conditions: 0${}^{\circ }$ and $\pm 6^{\circ }$ preswitch heading angles, ±3, $\pm 6^{\circ }$, $\pm 12^{\circ }$ switch angles. We generated 10 repetitions of each condition; only the initial placement of dots in the scene varied randomly between them. We digitized each sequence (“optic flow sample”) into 34 frames at 128 × 128 pixel resolution. The switch occurred at frame 30. The simplified version of the CD model operates directly on the optic flow field at every frame of each video sample (Layton et al., 2012). Therefore, the shape of each sample processed by the model is 128×128×2×34, where 2 refers to the horizontal and vertical components of the optic flow vector field at every frame.

**Figure 2.**
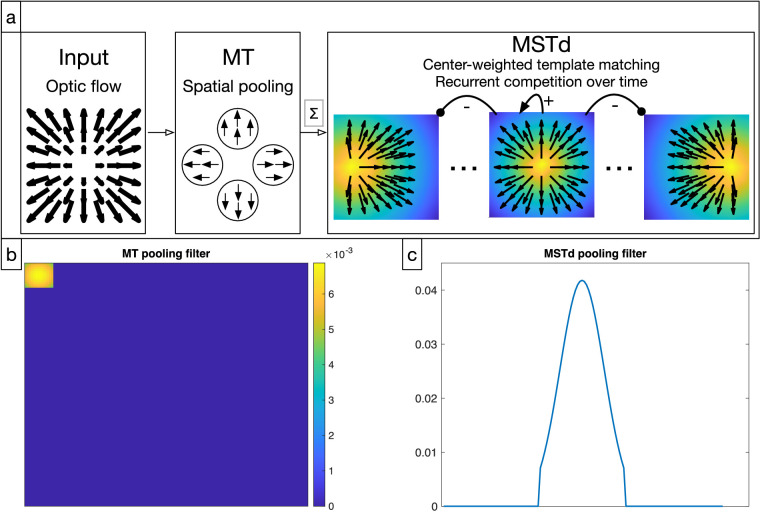
Overview of the CD model stages. (a) The model takes as input a temporal sequence of optic flow vector fields (left). At each time the model MT stage (middle) pools the motion signal, which spatially disperses the optic flow components. The resulting vector field is matched with a set of radial optic flow templates, each with a different preferred heading direction. Neurons compete with one another over time through recurrent connections: each cell enhances its own heading signal while inhibiting the others. (b) Two-dimensional Gaussian filter representing the receptive field of one MT unit shown to scale relative to the model representation of the visual field. (c) One-dimensional Gaussian pooling filter used in model MSTd shown to scale relative to the horizontal extent of the visual field.

As shown in Figure 2a, the first stage of the model corresponds to area MT. In the simplified CD model of Layton et al. (2012), MT spatially disperses optic flow vectors by convolving the optic flow horizontal and vertical components separately with a Gaussian filter (Figure 2b) specified by $r_{MT}$, $\sigma_{MT}$ in Table 1. This spatial pooling operation coarsely models the subpopulation of MT neurons without suppressive surrounds (Born, 2000; Born & Bradley, 2005; Yu et al., 2018).

The next model stage corresponds with the area MSTd, where the spatially pooled output of model MT is matched against a set of optic flow templates tuned to radial expansion with different FoE positions (Figure 2, right panel). Each template is represented by a filter that is maximally sensitive to the heading that coincides with its FoE position. As in Layton et al. (2012), we generate templates with FoE positions sampled at every pixel along the horizontal midline of the optic flow field. Each template matches the model MT signal (shape: 128×128×2) with the templates (shape: $128\times 128\times N_{\text{temps}}\times 2$) in a dot product operation to yield $N_{\text{temps}}\times$1 activations, where $N_{\text{temps}}$ corresponds with the number of templates. Each template is weighted by the inverse distance from the FoE to increase selectivity to the FoE (see Layton et al., 2012, for details). The raw template activations are smoothed by a one-dimensional Gaussian filter (Figure 2c) specified by $r_{MST}$, $\sigma_{MST}$ in Table 1. The smoothed template activations enter into a competitive network where each neuron uses recurrent on-center/off-surround connections to enhance the heading signal to which it is tuned over time while suppressing weaker heading signals.

We used a population vector approach to decoding heading rather than the winner-take-all method used by Layton et al. (2012). We made this change so that every neuron contributes to the heading estimate, not only the neuron that achieves the maximal activation. If $x_{i}$ represents the activation of the MSTd neuron tuned to heading $h_{i}$ the population vector heading estimate $i^{*}$ is computed according to:

(3) $$\begin{array}{c} i^{*}=\frac{\sum_{i=1}^{N_{\text{temps}}}x_{i}h_{i}}{\sum_{i=1}^{N_{\text{temps}}}x_{i}}\; \end{array}$$

As in the main experiment, we assessed the heading bias, the differential error that the model produced on switch and no-switch trials (Equation 2).

We selected the model parameters in Table 1 by random search. On each iteration of the search, we sampled a value for each parameter listed in Table 1 within a respective predefined range (described elsewhere in this article). We instantiated the model with the sampled parameter values and used the model to process all optic flow samples from the experiment. Then we created a plot of the mean error in the model heading estimates and compared it by visual inspection to the corresponding plot of the human data. If there was a close match, we stopped the search and recorded the sampled parameter values. If there was not a close match, we repeated the search for another iteration. Only a few search iterations were required to find parameter values that yield estimates that closely resembled human judgments.

We used known neurophysiology and existing models to inform the ranges over which we searched for parameters where possible. We searched for the MT RF radius ($r_{MT}$) over $\left[2^{\circ },12.5^{\circ }\right]$, which matches the range provided by Felleman and Kaas (1984) and is compatible with the broader literature (Deangelis & Uka, 2003; Born & Bradley, 2005). In MSTd, the RF sizes span a considerable range, reaching more than 100${}^{\circ }$ (Tanaka et al., 1986). Simulations reveal that the accuracy of heading estimates improves when the RF size of model MSTd units increases, though the improvement plateaus once units have a RF radius of about $21.5^{\circ }$ (Yumurtaci & Layton, 2021). With this constraint in mind, we sampled the value of $r_{MSTd}$ over $\left[7.5^{\circ },22^{\circ }\right]$. The passive decay rate parameter (A) controls how quickly unit activation decays to zero in the absence of input. We searched for values over $\left[0.1,1.0\right]$, given that prior models of optic flow processing tend to use a value in this range (Yumurtaci & Layton, 2021; Layton et al., 2012). The exponential moving average constant c (0≤c≤1) determines the degree to which MSTd activations from the recent time history influence the current activation. We sampled values for c over $\left[0.2,0.8\right]$ to prevent extreme cases where the activation is determined exclusively by the past or current optic flow signal. We sampled the remaining parameters over broad ranges ($\sigma_{MT}$: $\left[0.1^{\circ },28^{\circ }\right]$, $\sigma_{MSTd}$: $\left[0.1^{\circ },35^{\circ }\right]$, B: $\left[1,150\right]$).

### Results and discussion

#### Mean bias in heading judgments

**Figure 3.**
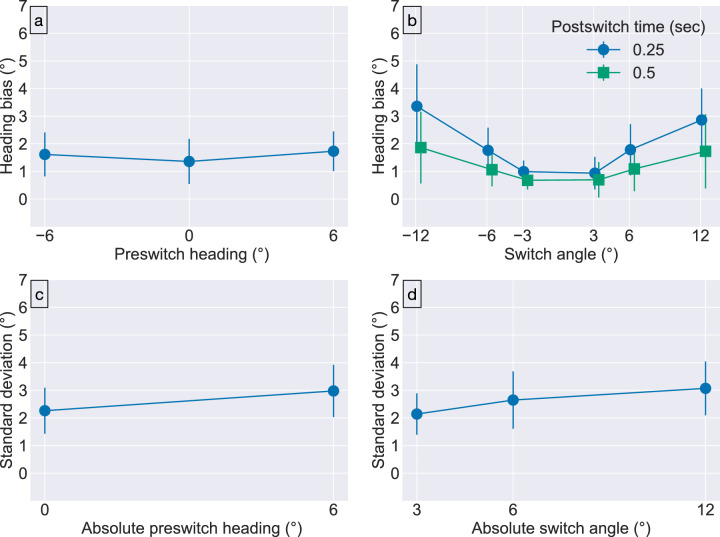
Experiment 1 results. (top row) Mean heading bias for the (a) preswitch heading directions and (b) switch angles. Positive values indicate bias toward the preswitch heading (attractive effect). (bottom row) Precision of heading judgments represented as the standard deviation across repetitions of the same condition. Error bars show 95% CIs and correspond to the variability across subjects in the mean and standard deviation parameter estimates. The horizontal position of plot markers in (b) is displaced slightly for visual clarity.

The primary aim of Experiment 1 was to investigate whether the optic flow associated with the preswitch heading influenced perception of the postswitch heading. Figure 3a shows the bias in heading judgments of the postswitch heading obtained for the different switch angles. In the plot, positive heading bias indicates bias in judgments toward the preswitch heading compared with comparable trials that did not contain a switch (Figure 1b), zero indicates no effect of the heading switch, and negative heading bias indicates bias away from the preswitch heading (Figure 1c). From Figure 3a, it is apparent that the positive mean bias and 95% confidence intervals (CIs) that do not include 0${}^{\circ }$ for each preswitch heading (M ± CI; −6${}^{\circ }$: 1.62 ± 0.80${}^{\circ }$, 0${}^{\circ }$: 1.36 ± 0.82${}^{\circ }$, 6${}^{\circ }$: 1.73 ± 0.72${}^{\circ }$). This reveals that the preswitch heading exerts an attractive effect on judgments when heading changes during the trial. This result is consistent with a visual mechanism that accumulates evidence about heading and takes time to shift the evolving estimate.

We analyzed the mean heading bias with respect to the independent variables using a three-way repeated measures ANOVA (6 switch angles × 3 preswitch headings × 2 postswitch durations). The analysis revealed a significant main effect of preswitch heading, F(2, 22) = 5.746, p = 0.010, $\eta_{p}^{2}=0.343$, a significant main effect of switch angle, F(1.3, 14.34) = 14.525, p = 0.001, Greenhouse-Geisser corrected, $\eta_{p}^{2}=0.569$, and a significant interaction between switch angle and postswitch duration, F(5, 55) = 8.677, p < 0.001, $\eta_{p}^{2}=0.441$. The significant interaction prompted us to follow up with separate one-way repeated measures ANOVAs for the two postswitch durations (Figure 3b). We found a significant effect of switch angle for both 250-ms, F(1.39, 15.26) = 19.284, p < 0.001, Greenhouse-Geisser corrected, $\eta_{p}^{2}=0.637$, and 500-ms, F(1.58, 17.34) = 7.807, p = 0.006, Greenhouse-Geisser corrected, $\eta_{p}^{2}=0.415$, postswitch durations. To examine whether the mean bias increases with switch angle, we collapsed across switch angle sign and performed Tukey honest significant difference (HSD) post hoc tests on the bias within each postswitch duration group. For the 250-ms postswitch duration, there was a significant difference between the 12${}^{\circ }$ absolute switch angle and the other absolute switch angles ( 3${}^{\circ }$ vs. 12${}^{\circ }$: p < 0.001, 6${}^{\circ }$ vs. 12${}^{\circ }$: p = 0.003). There was no significant difference between the 3${}^{\circ }$ and 6${}^{\circ }$ conditions (p = 0.120). For the 500-ms postswitch duration, there was only a significant difference between the 3${}^{\circ }$ versus 12${}^{\circ }$ absolute switch angles (3${}^{\circ }$ vs. 12${}^{\circ }$: p < 0.025, 6${}^{\circ }$ vs. 12${}^{\circ }$: p = 0.208, 3${}^{\circ }$ vs. 6${}^{\circ }$: p = 0.632). This analysis indicates an increase in bias with absolute switch angle for both postswitch durations, at least between the smallest and largest absolute switch angles considered here.

A key prediction was that extended viewing time of the postswitch optic flow should decrease heading bias owing to the preswitch heading. A paired samples, one-tailed *t* test reveals that the mean bias across subjects was significantly lower for the 500-ms postswitch duration than the 250-ms postswitch duration (t(11) = −5.524, p< 0.001, Cohen’s d = 1.590), which supports the prediction. Figure 3b prompted us to examine whether the significantly reduced bias in the case of the 250-ms postswitch duration holds for different switch angles. Post hoc one-tailed *t* tests (using the Holm correction to adjust p) indicated that the bias with the 500-ms postswitch duration was significantly smaller than the 250-ms postswitch duration for all but the 3${}^{\circ }$ switch angle (−12${}^{\circ }$: p = 0.001, −6${}^{\circ }$: p = 0.017, −3${}^{\circ }$: p = 0.045, 3${}^{\circ }$: p = 0.100, 6${}^{\circ }$: p = 0.0496, 12${}^{\circ }$: p = 0.011). This provides evidence that longer viewing durations of the postswitch optic flow improves the accuracy of heading judgments for the larger switch angles. Because there is less of a difference between the preswitch and postswitch optic flow fields for the 3${}^{\circ }$ switch angle, perhaps the visual system requires longer than 500-ms of postswitch optic flow to garner more accurate judgments. In these cases, however, the mean bias for either postswitch time is already quite small (1${}^{\circ }$ or less).

#### Mean precision of heading judgments

Next, we examined the precision of postswitch heading judgments. Following Cuturi and Macneilage (2013), we define precision as the standard deviation of each subject’s heading judgments computed across the five repetitions of each condition. To focus on the influence of eccentricity on the precision of heading judgments, we collapsed the standard deviations across preswitch and switch angle sign. We analyzed the standard deviations using a three-way repeated measures ANOVA (3 absolute switch angles × 2 absolute preswitch headings × 2 postswitch durations). The analysis resulted in a significant main effect of absolute preswitch heading, F(1, 11) = 21.520, p< 0.001, $\eta_{p}^{2}=0.662$, and a significant main effect of absolute switch angle, F(2, 22) = 6.323, p = 0.007, $\eta_{p}^{2}=0.365$. Figure 3c shows that the significant main effect of absolute preswitch heading corresponds to a decrease in the mean precision as absolute preswitch heading increases from 0${}^{\circ }$ to 6${}^{\circ }$. We performed Tukey HSD post hoc tests to examine whether the precision decreases as the absolute switch angle increases (Figure 3d). There was a significant difference in the precision between the 3${}^{\circ }$ and 12${}^{\circ }$ absolute switch angle conditions (p < 0.001), but not between the other pairs of conditions (3${}^{\circ }$ vs. 6${}^{\circ }$, p = 0.107; 6${}^{\circ }$ vs. 12${}^{\circ }$, p = 0.210). This analysis indicates a decrease in mean precision, at least from the smallest to largest absolute switch angles considered.

#### No-switch trial duration

Because the duration of no-switch trials matched that of the postswitch period of Switch trials, the total duration of no-switch trials was 750-ms shorter than that of switch trials. We performed a control experiment with three subjects to examine whether the reduced trial duration in the no-switch condition could explain the influence of the preswitch heading. The design of the control experiment matched that of Experiment 1, except that we introduced a 750-ms blackout period at the beginning of no-switch trials to equate the trial duration of no-switch and switch trials. We obtained the same pattern of results as in Experiment 1, which indicates that the shorter no-switch trial duration does not mediate the influence of the preswitch heading.

#### Center bias

Humans generally underestimate the heading angle when judging heading from optic flow. This phenomenon is known as “center bias” because underestimation implicates error toward the center of the screen (Sun et al., 2020; Xing & Saunders, 2016; Warren & Saunders, 1995; Layton & Fajen 2016c, b; Royden & Hildreth, 1996; Warren et al., 1988). Indeed, humans underestimated heading in Experiment 1—overall judgments were 68.83% ± 25.30% (mean ± 95% CI) of the true heading angle. Although the preswitch heading did in some cases appear toward the center of the postswitch heading, center bias is an unlikely explanation of our findings for several reasons. First, our analysis considers the difference in heading judgments between switch and no-switch trials that possess the same heading prior to judgment (heading bias; Equation 2). The heading bias metric, therefore, factors out any bias due to a center screen effect, which should be the same within each matched condition. Second, in cases where the preswitch heading lies peripheral to the postswitch heading, judgments exhibit peripheral bias compared with matched no-switch trials—toward the preswitch heading rather than the center (switch from −6${}^{\circ }$ to −3${}^{\circ }$ vs. −3${}^{\circ }$ no-switch: t(11) = 2.520, p = 0.014, Cohen’s d = 0.728; switch from −6${}^{\circ }$ to 0${}^{\circ }$ vs. 0${}^{\circ }$ no switch: t(11) = 2.759, p = 0.009, Cohen’s d = 0.797; switch from 6${}^{\circ }$ to 3${}^{\circ }$ vs. 3${}^{\circ }$ no-switch: t(11) = 3.603, p = 0.002), Cohen’s d = 1.040; Switch from 6${}^{\circ }$ to 0${}^{\circ }$ vs. 0${}^{\circ }$ no switch: t(11) = 4.675, p < 0.001, Cohen’s d = 1.350).

#### Comparison with CD model predictions

**Figure 4.**
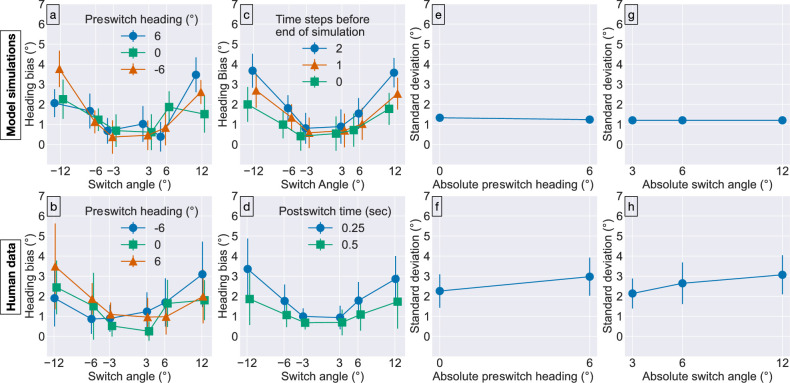
Comparison of CD model simulations (top) with human heading judgments from Experiment 1 (bottom). All error bars show 95% CIs. Error bars in plots of model performance represent variability in mean and standard deviation parameter estimates across repetitions of the same experimental condition. Error bars in plots of human performance capture variability in parameter estimates across subjects. The horizontal position of plot markers in (a–d) is displaced slightly for visual clarity.

To more precisely quantify the predictions of the CD model and compare them with human heading judgments, we simulated the model with the conditions from Experiment 1 (see Methods for details). We begin by considering the prediction that estimates of the postswitch heading should depend on the preswitch heading. Figure 4a shows that the bias in model estimates of the postswitch heading for the different switch angles and preswitch heading directions are in close agreement with humans judgments (Figure 4b).

Next, we examined the prediction that the accuracy of heading perception should improve over time. We quantified this by examining the model heading estimate at several simulation time steps before the end of each optic flow sample. Time steps closer to the end of the simulation correspond to longer times since the switch. Figure 4c demonstrates a temporal dependence in the bias that is similar to that in the human judgments (Figure 4d).

Finally, we explored whether the CD model captures the mean precision of human heading judgments. As with the human judgments, we quantified the precision by computing the standard deviation of model heading estimates across repetitions of each condition. Figure 4e, g show that the standard deviation of model estimates is approximately $1^{\circ }$ for each of the absolute preswitch heading and switch angle conditions, respectively. This is lower than the 2${}^{\circ }$ to 3${}^{\circ }$ mean standard deviation of human judgments in corresponding conditions (Figure 4f, h). One factor that may account for the discrepancy with the human data is sampling of heading tuned units in model MSTd. For simplicity, we have configured the model to contain units tuned to regularly spaced headings along the horizontal axis. However, neurophysiological evidence indicates that heading tuning in primate MSTd may be biased toward the periphery (Takahashi et al., 2007; Gu et al., 2010). Simulations performed by Yumurtaci and Layton (2021) show that introducing a weak peripheral bias to heading tuning in model MSTd increases the standard deviation of central heading estimates to approximately 2${}^{\circ }$ to 3${}^{\circ }$, which would bring the model precision in agreement with that of humans.

In summary, the CD model produces a pattern of heading bias that is similar to that of humans. Both human judgments and model estimates exhibit an attractive influence of the preswitch heading. Bias decreases when the postswitch optic flow appears for a longer period of time in the case of most of the switch angles tested. The model estimates exhibit greater precision than human judgments. Taken together, this shows that a mechanism that accumulates evidence about heading over time like the one in the CD model is capable of accounting for the human data.

## Experiment 2: Periods of blackout

**Figure 5.**
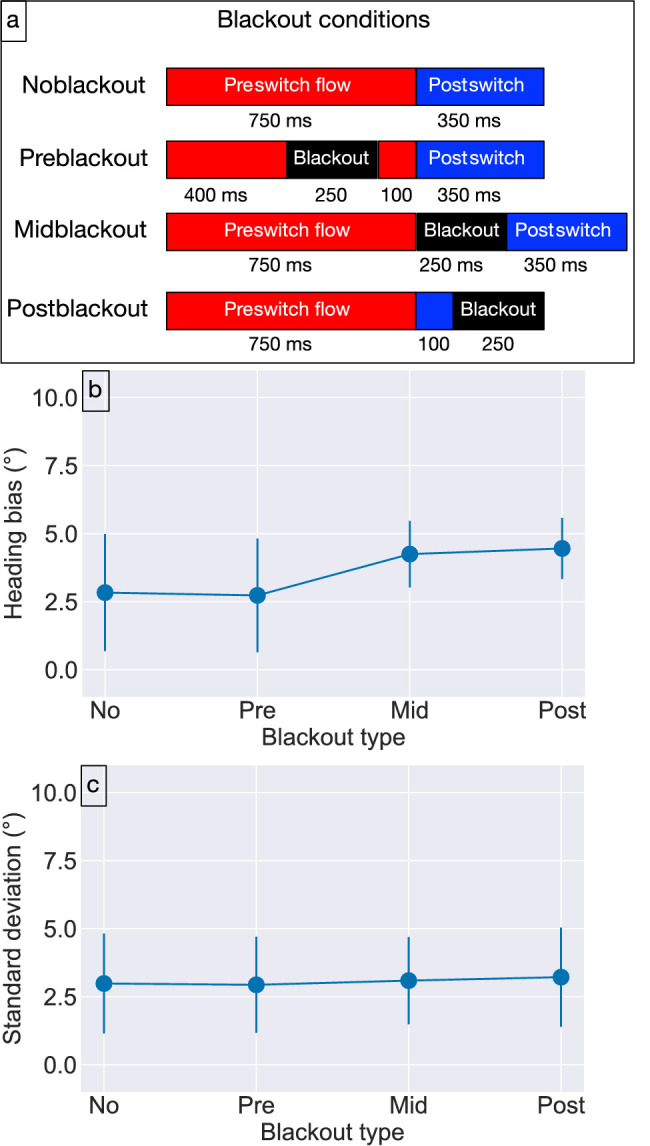
Experiment 2 blackout condition schematic (a), and results (b and c). (a) All durations (lengths of bars) have units of milliseconds. Lengths of bars are not scaled to reflect exact durations. (b) Mean bias in human heading judgments in the different blackout conditions. (c) Mean precision of human heading judgments in the different blackout conditions. Error bars show 95% CIs and correspond with the variability across subjects in the mean and standard deviation parameter estimates.

Experiment 1 presents evidence that the duration for which optic flow is viewed after the heading switch influences the accuracy of postswitch heading judgments. We explored the role of optic flow duration around the heading switch event in more detail with Experiment 2. Specifically, we introduced a 250-ms blackout period during which the optic flow temporarily ceases before the switch (“preblackout condition”), just as the switch occurs (“midblackout condition”), and after the switch (“postblackout condition”). Figure 5a summarizes the different blackout conditions (see also Movies S3–S5). In the case of the preswitch and postblackout conditions, the blackout diminished the viewing duration of the preswitch and postswitch optic flow, respectively. In contrast, the midblackout condition did not reduce the viewing duration of either period and added 250-ms to the overall trial length. If the absence of optic flow weakens any evolving heading estimate that may exist, the blackout should decrease the bias in the preblackout and midblackout conditions. On the other hand, the bias should increase in the postblackout condition because with an evolving estimate and the reduced postswitch optic flow duration, judgments should mostly reflect the preswitch optic flow.

### Methods

#### Participants

Thirteen college students between the ages of 18 and 21 years participated in the study for monetary compensation. All were naïve with respect to the purpose of the experiment, and had normal or corrected-to-normal vision. None of the subjects participated in Experiment 1. We obtained written consent from all participants before the experiment started. The experimental protocol was approved by the Institutional Review Board at Colby College and is in compliance with the Declaration of Helsinki.

#### Visual stimuli

The visual stimuli only differed modestly from those used in Experiment 1. On trials without the blackout (“noblackout” condition), we fixed the preswitch and postswitch optic flow durations to 750 and 350-ms, respectively. In each blackout condition, subjects viewed an entirely black screen during the 250-ms blackout period. Individual trials had at most one blackout period. As Figure 5a shows, the blackout in the preblackout condition ended 100-ms before the heading switch (400–650-ms relative to trial onset). In the midblackout condition, the blackout separated the preswitch and postswitch optic flow. In the postblackout condition, the blackout followed the first 100-ms of the postswitch optic flow (850–1100-ms relative to trial onset). Because this means that the blackout would persist through the end of the trial, we added approximately three frames (50-ms) of self-motion consistent with the postswitch heading after the blackout. This ensured that subjects did not judge their heading in darkness in the postblackout condition and made the conditions under which subjects judged their heading comparable to the other trial types.

In the preblackout and postblackout conditions, the blackout removed 250-ms of optic flow from the preswitch and postswitch optic flow, respectively. By contrast, the trial duration was extended by 250-ms in the midblackout condition to account for the blackout period.

#### Procedure

The procedure was the same as in Experiment 1. Each subject completed 415 trials of the experimental conditions blocked by repetition (5) in a fully crossed design. Each block consisted of 72 trials with a switch (3 preswitch heading angles × 6 switch angles × 4 blackout conditions) and 11 no-switch trials (11 unique postswitch headings). No feedback was provided in the practice task or main experiment. The experiment lasted approximately 60 minutes.

#### Analysis

We used the same analysis as in Experiment 1.

#### CD model simulations

We simulated 32 rather than the 34 frames of optic flow in the Experiment 1 simulations to match the proportional reduction in postswitch optic flow time in Experiment 2. As in the simulations for Experiment 1, the heading changed at frame 30 on Switch trials. We simulated the effect of blackout by removing the optic flow input for one time step (frame of the optic flow sequence). The MSTd activation during this time depended exclusively on recurrent on-center/off-surround dynamics and the evolving signal that developed from optic flow before the blackout. To simulate the preblackout, midblackout, and postblackout conditions, we started the blackout period on frames 28, 30, and 31, respectively. This meant that one frame of optic flow separated the switch and the blackout in both the preblackout and postblackout conditions. As in the main experiment, the postblackout condition reduced the postswitch optic flow duration, whereas the midblackout condition did not. We examined the heading bias produced by the model after time step 32 (i.e., frame 32), which meant that the postswitch optic flow appeared for one time step in the postblackout condition and three time steps in the other conditions. Our goal with these simulations was to demonstrate qualitative model predictions in each blackout condition. We did not attempt to simulate the exact time course of each experimental trial.

### Results and discussion

#### Mean bias in heading judgments

Given symmetric performance for left and right switch angles, we collapsed across switch angle sign. A three-way repeated measures ANOVA (3 preswitch heading angles × 3 absolute switch angles × 4 blackout conditions) revealed significant main effects of preswitch heading, F(2, 24) = 3.478, p = 0.047, $\eta_{p}^{2}=0.225$, switch angle, F(1.04, 12.52) = 14.499, p = 0.002, Greenhouse-Geisser corrected, $\eta_{p}^{2}=0.547$, and blackout type, F(1.12, 13.46) = 4.919, p = 0.041, Greenhouse-Geisser corrected, $\eta_{p}^{2}=0.291$. The significant main effects of preswitch heading and switch angle reproduce the attractive influence of the preswitch heading from Experiment 1.

To interpret the significant main effect of blackout type, we plotted the mean heading bias within each blackout condition (Figure 5b). The elevated mean bias in the midblackout and postblackout conditions (right two plot markers) compared with the noblackout and preblackout conditions (left two plot markers) suggests that a blackout later in the trial results in increased bias toward the preswitch heading compared with when the blackout appears early in the trial or is absent. We performed Tukey HSD post hoc tests to analyze the relative influence of blackout type on heading judgments. There was no significant difference between the noblackout (2.83° ± 2.15${}^{\circ }$) and preblackout (2.73° ± 2.09${}^{\circ }$) conditions (noblackout vs. preblackout: p = 1.000) as well as the midblackout (4.25° ± 1.22${}^{\circ }$) and postblackout (4.26° ± 1.13${}^{\circ }$) conditions (midblackout vs. postblackout: p = 1.000). Although the differences between the later blackout conditions (midblackout and postblackout) and the noblackout condition did not reach significance (noblackout vs. midblackout: p = 0.055, no-blackout vs. postblackout: p = 0.053), differences with respect to the preblackout condition were significant (preblackout vs. midblackout: p = 0.033, preblackout vs. postblackout: p = 0.032). This analysis indicates that there is no significant difference in the mean heading bias when the blackout is absent and when the blackout appears shortly before the switch. The presence of a blackout at the time of the switch or later increases the heading bias toward the preswitch heading.

#### Mean precision of heading judgments

Figure 5c shows that the mean precision in each blackout condition (approximately $3^{\circ }$) is comparable with the (approximately 2° to 3${}^{\circ }$) garnered in Experiment 1. A three-way repeated measures ANOVA (3 preswitch heading angles × 3 absolute switch angles × 4 blackout conditions) on the standard deviation computed across repetitions of each condition revealed significant a main effect of preswitch heading, F(1.4, 16.82) = 4.072, p = 0.048, Greenhouse-Geisser corrected, $\eta_{p}^{2}$ = 0.253. The mean precision of human judgments did not vary significantly across the blackout conditions.

#### Comparison with CD model predictions

The lack of a significant difference between the noblackout and preblackout conditions (Figure 5b) indicates that the 250-ms interruption in the preswitch optic flow did not affect the dependence of the preswitch heading on heading judgments. A fast, robust rebound in heading signals during the 100-ms of preswitch optic flow that follows the blackout (Figure 5a) could explain this finding. However, if 100-ms of optic flow were sufficient for neural signals to attune to the present heading more generally, it is unclear why judgments would exhibit any bias toward the preswitch heading in any condition.

**Figure 6.**
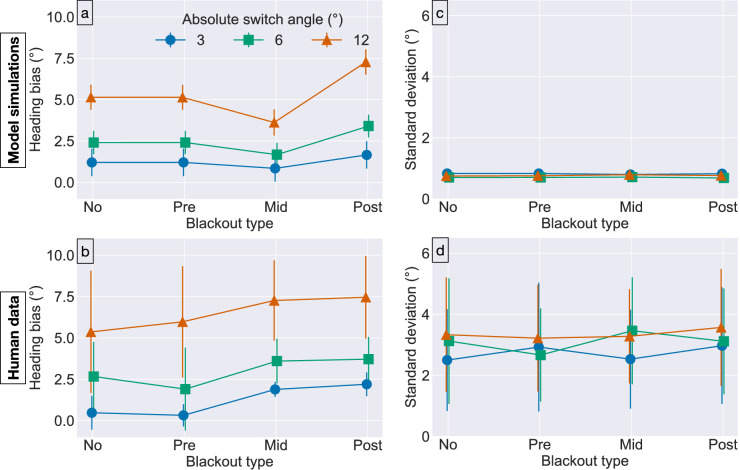
Comparison of CD model simulations (top) with human heading judgments from Experiment 2 (bottom). All error bars show 95% CIs. Error bars in plots of model performance represent variability in mean and standard deviation parameter estimates across repetitions of the same experimental condition. Error bars in plots of human performance capture variability in parameter estimates across subjects. The horizontal position of plot markers is displaced slightly for visual clarity.

One possible explanation is a temporally evolving heading signal that persists throughout the blackout period. This would account for the lack of a difference between the noblackout and preblackout conditions as well as the increased bias in the postblackout condition due to an enduring influence of the preswitch heading. We simulated the CD model with each blackout condition to determine more precisely, whether its evolving heading signal could capture the human data. Figure 6a plots the bias of model estimates in each blackout condition from Experiment 2, broken down by absolute switch angle, and Figure 6b shows the bias of human judgments in the corresponding conditions. The CD model captures the effect of switch angle and reproduces the increased heading bias in the postblackout condition compared with the preblackout and noblackout conditions. The difference in bias emerges in the model because the brief postswitch optic flow duration is not sufficient for the sensory signal to overtake the evolving signal that mainly reflects the preswitch heading. Consistent with the human data, the bias produced by the model in the noblackout condition is no different than in the preblackout conditions.

It is noteworthy that the CD model does not reproduce the elevated bias in the midblackout condition (Figure 6b). Increasing the postswitch optic flow duration mitigates the bias in the CD model, whereas the same does not occur in the human judgments—the postblackout and midblackout conditions include different postswitch optic flow durations, 100-ms versus 350-ms, respectively, yet yield similarly elevated bias. This suggests that postswitch optic flow duration alone does not underlie the elevated bias in both conditions. For extended discussion related to this discrepancy between the model results and human data, please see the General discussion.

Figures 6c and d show that human judgments and model estimates both demonstrate comparable mean precision to that obtained in Experiment 1. That is, the model estimates once again exhibit greater mean precision than the human judgments.

In summary, Experiment 2 offers insight into the role of optic flow around the time of the heading switch event. While a blackout shortly before the switch did not affect human judgments, a blackout shortly after the switch until the time of judgment increased heading bias toward the preswitch heading. These findings support evidence from Experiment 1 that longer postswitch optic flow durations decrease heading bias, which could be explained by an evolving heading signal that persists throughout the blackout period, as demonstrated by the CD model simulations. However, this explanation does not capture the elevated bias in the midblackout condition. A mechanism not included in the model may be involved.

## General discussion

We investigated the stability of human heading perception as heading changes during the trial. Across two experiments, we found that the heading direction before the switch influenced judgments of the heading after the switch. Judgments fell in between the preswitch and postswitch headings and demonstrated bias toward the preswitch heading. This attractive influence of the preswitch heading is compatible with the predictions of the CD model, which we confirmed through simulations of the experimental conditions. Because the model explains the results through competitive interactions among heading sensitive neurons that unfold over time, another prediction that emerges is that the accuracy of heading perception should improve over time. Our data are largely consistent with this prediction, although see forthcoming discussion of the midblackout condition in Experiment 2. Overall, our findings are compatible with a mechanism that accumulates evidence from optic flow to stabilize heading perception over time.

Our study does not address whether such an evidence accumulation process may depend on an instantaneous vector field representation of optic flow or higher-order components, such as optic acceleration. This is because our stimuli do not limit the availability of certain optic flow components. The version of the CD model simulated here processes a sequence of optic flow vector fields over time and accounts for the pattern of human heading bias, which suggests that the velocity component of optic flow may be sufficient to capture human judgments in our task. It is noteworthy, however, that Burlingham and Heeger (2020) found that the availability of higher-order optic flow, but not velocity alone, was necessary to support accurate heading perception in their experiment. Their stimulus contains rotational optic flow, whereas ours does not, so it is possible that higher-order optic flow may play a role when estimating heading in the presence of rotation.

### Stability of heading perception

Humans produce errors when judging heading from optic flow that are small in the center and grow with eccentricity (Yumurtaci & Layton, 2021; Sun et al., 2020; Cuturi & Macneilage, 2013; Van Den Berg, 1992; Warren et al., 1988). These errors tend to demonstrate bias toward the center of the screen rather than symmetry about the true heading direction (Xing & Saunders, 2016; Sun et al., 2020; Layton & Fajen, 2016c; Warren et al., 1988; Royden & Hildreth, 1996; Warren & Saunders, 1995). In virtually all studies of human heading perception from optic flow, individual trials involve simulated self-motion along a heading direction that is drawn randomly from a symmetric distribution about the straightahead. When faced with uncertainty it makes sense for subjects to orient judgments toward to the mean of the empirical heading distribution—the center of the screen. From a Bayesian perspective, the center of the screen could be viewed as a useful prior, because it minimizes the average discrepancy to most of the headings. When heading changes within the trial, however, the center of the screen may serve as a less useful reference, especially when the postswitch heading is proximal to the preswitch heading as it was in our study. The preswitch heading may represent a more reliable reference since it represents the last known heading and dominates the visual experience so far in the trial. The distinction between bias toward the center of the screen and the preswitch heading under different circumstances raises the intriguing possibility that humans attune their judgments to recent viewing statistics when faced with uncertainty. Such adaptation would contribute to the stability of heading perception when self-motion may suddenly change, when sensory signals are noisy or undergo flux without actual changes to self-motion, and through brief interruptions to sensory signals (e.g., temporary blackouts).

### Blackouts and heading perception

In Experiment 2, human heading judgments exhibited comparable bias in the midblackout and postblackout conditions, even though the duration of optic flow differed after the blackout (Figure 5a). Thus, the viewing duration of the postswitch optic flow cannot explain the elevated bias. The CD model captures the human bias in the postblackout condition, but the bias in model estimates is too weak in the midblackout condition. This occurs because that network activation to the preswitch heading does not propagate through the blackout with sufficient strength to yield human-like bias. The extent of temporal evolution in the model could be lengthened (e.g., via parameters A and c) to better allow the activation to persist throughout the blackout and thereby increase the bias in the midblackout condition. Such a change, however, would also increase the bias in the noblackout condition because the two conditions share the same preswitch and postswitch optic flow durations. This would place the model estimates in conflict with the human bias in the noblackout condition. The same problem would emerge with the preblackout condition. Taken together, this analysis suggests that the CD model cannot account for the human bias in the midblackout because it lacks a crucial mechanism.

One property that distinguishes the midblackout condition from the others is that the heading before and after blackout period does not match (Figure 5a). Owing to this uncertainty, let us assume that subjects attempt to anticipate the postswitch heading during the blackout. Although the postswitch heading may be within $\pm 12^{\circ }$ of the preswitch heading, the average switch angle is 0${}^{\circ }$ owing to symmetry. Note that anticipating a switch angle that is different than 0${}^{\circ }$ would occasionally result in large errors in prediction. For example, anticipating a 3${}^{\circ }$ switch when it actually is −3${}^{\circ }$ would result in a prediction error of 6${}^{\circ }$ instead of only 3${}^{\circ }$ if 0${}^{\circ }$ were anticipated. Therefore, it is reasonable for subjects to anticipate that the forthcoming heading will coincide with the one experienced before the blackout. If the visual system requires additional time to resolve mismatches between the anticipated and actual forthcoming headings, this could explain the elevated heading bias in the midswitch condition. In contrast, the visual system may not require additional time when there is a match, because the forthcoming heading would affirm the expectation. This would explain why the preblackout condition did not yield elevated heading bias. The issue of expectation may not bear on judgments in the postblackout condition since the trial terminates immediately after the blackout and any expectation would nether affirmed nor violated. Indeed, the CD model captures the elevated human bias in the postblackout condition without explicitly modeling expectation (Figure 6a). Dynamics that depend on whether the optic flow signal affirms or violates expectations about the heading direction would be compatible with adaptive resonance theory (Grossberg, 1976, 2013; Brito Da Silva, Elnabarawy, & Wunsch, 2019; Layton, 2021) and predictive coding (Friston, 2010).

Spatial attention represents one means by which expectations could shape the temporal dynamics of heading perception. MSTd neurons fire more weakly (Dubin & Duffy, 2007) and yield peak activity that is delayed by an average of 177-ms (Dubin & Duffy, 2009) when the attended location is far from the FoE compared with when it is near. Layton and Browning (2012) show that the CD model accounts for the spatiotemporal effects of attention in the MSTd data of Dubin and Duffy (2007, 2009) when an attentional signal is embedded in the model. The reason is that it requires more time for a clear heading estimate (“winner”) to emerge from the competition among heading sensitive cells when the optic flow and attentional signals are spatially offset. In contrast, optic flow and attentional signals that overlap substantially reinforce one another, which speeds up the dynamics. If subjects attend the location of the last seen FoE during the blackout, the neural data of of Dubin and Duffy (2007, 2009) and simulations of Layton and Browning (2012) suggest that it may take longer in the midblackout condition for the visual system to accurately estimate the postswitch heading, especially when the switch angle is large. Indeed, Figure 6b shows that the main source of the elevated bias in Experiment 2 compared with Experiment 1 comes from the largest switch angle tested ($\pm 12^{\circ }$).

### Extent of temporal integration when processing optic flow

The evolving influence of the preswitch heading in the present study is consistent with findings from other psychophysical experiments on the temporal dynamics in optic flow processing. In the experiments of Duijnhouwer, Krekelberg, Van Den Berg, and Van Wezel (2010), subjects judged the singularity position (FoE in the case of a radial expansion pattern) in the optic flow field that appeared while tracking a moving target with a pursuit eye movement. The singularity displaced over time due to the addition of the rotational optic flow component contributed by the eye movement as well as the changing perspective. Duijnhouwer et al. (2010) show that the pattern of errors made in locating the singularity are consistent with a temporal integration model (leaky integrator) that gives more weight to more recently viewed retinal singularity positions in each optic flow stimulus. Interestingly, the 500-ms time constant parameter in the best fitting model implicates a fairly long integration window, which agrees with the improvements in MSTd neuron pattern discrimination thresholds over 1 s (Heuer & Britten, 2004) and the estimated approximately 430-ms required for human subjects to view optic flow before being capable of accurately saccading to the FoE at a 15${}^{\circ }$ eccentricity with respect to a central fixation point (Hooge, Beintema, & Van Den Berg, 1999).

Sikoglu, Calabro, Beardsley, and Vaina (2010) provide support for the use of temporal integration in human heading perception. Their experiment focuses on the influence of different types on noise on human heading discrimination thresholds. Evidence for temporal integration emerges by comparing human performance in their random-walk noise condition, where random perturbations corrupt the 3D trajectory of each dot independently on each frame, and their fixed trajectory condition, where a single directional perturbation affects the trajectory of each dot over the entire trial. The visual system may in principle use spatiotemporal integration to recover heading in the random walk noise condition because the noise is unbiased over time, but temporal integration should not benefit heading estimates in the fixed trajectory condition because the dots do not move consistently with heading at any point throughout the trial. Indeed, subjects judged heading less accurately in the fixed trajectory condition, suggesting that the visual system leverages temporal integration to support heading perception. An analysis performed by Sikoglu et al. (2010) shows that subject performance can be explained by optic flow integration over a 200-ms window.

An integration window over at least several hundred milliseconds agrees with the results of Layton and Fajen (2016c), which show progressive improvement in the accuracy of human heading judgments as the optic flow viewing duration lengthens over 150 to 1,500-ms that plateaus after approximately 500-ms. The present study supports this improvement in accuracy over time and generalizes it to cases where the heading direction changes (Figure 3b). Alefantis et al. (2022) show that the availability of optic flow over longer periods of time (500–1,500-ms) yields progressive improvements to human performance in an active steering and target interception task. These improvements may stem from more accurate heading perception since humans rely on heading to steer toward targets (Li & Cheng 2011a, 2013; Li & Niehorster, 2014).

Interestingly, longer optic flow viewing durations may not always accompany improved heading judgments. Grigo and Lappe (1999) studied the case of self-motion toward a single frontoparallel plane while horizontal (yaw) eye movements were simulated within the display. That is, human subjects fixated a stationary target centered on the computer screen and viewed optic flow that was generated by translating the virtual camera forward while rotating it to maintain gaze on a point located on the plane. Grigo and Lappe (1999) found that humans judged heading accurately in their 90${}^{\circ }$ × 90${}^{\circ }$ optic flow display when the viewing duration was short (400–500-ms), although, surprisingly, accuracy decreased when the optic flow was viewed over longer durations reaching 3.2 s. The decrease in accuracy coincides with subjects producing increasing amounts of bias over time toward the singularity in the optic flow, which does not correspond to the observer’s heading toward the plane because of the simulated camera rotation within the display. This finding is not necessarily at odds with ours showing that longer optic flow viewing durations after the switch improve the accuracy of postswitch heading judgments. The displays in the present study contain no rotation, so the accuracy of heading perception may only deteriorate in the presence of simulated rotation. This might be the case, because simulated rotation places the optic flow in conflict with the extraretinal information—the eye position of subjects remains fixed on the center of the screen, but the optic flow contains a rotational component that would naturally arise while moving the eye. Perhaps the visual system is capable of tolerating the conflict between optic flow and eye position over short durations, but the mechanism fails when the conflict grows too large or spans too much time.

### Flux in optic flow and the sensitivity of heading perception

Grigo and Lappe (1999) conducted an experiment wherein they presented subjects with the same 3.2-s simulated self-motion scenarios that garnered poor accuracy, but regenerated the dots every 400-ms. The stimulus was therefore identical to the 3.2-s versions except for the periodic random repositioning of dots. Fascinatingly, the accuracy of heading judgments improved substantially and judgments resembled those obtained from the short 400 to 500-ms durations. This suggests heading perception may benefit from the periodic resampling of the environment, which may be achieved under naturalistic conditions through eye and/or head movements. From this perspective, it is possible that the heading switch in the present study may have prevented a decline in sensitivity that might have otherwise occurred over longer periods of time.

Bossard, Goulon, and Mestre (2016) show that periodic change to optic flow may not only enhance heading perception, but perception of distance traveled along straight paths (path integration). They found that rhythmic oscillations in optic flow, including those that arise from head sway during walking, facilitate the perception of distance traveled when moving along a straight path compared to when none such oscillations arise. Thus, periodic flux to the optic flow could promote sensitivity not only to the direction of self-motion but also the speed of self-motion. Dynamic Bayesian modeling demonstrates qualitative shifts in how the speed of self-motion is estimated over time based on the buildup of uncertainty (Lakshminarasimhan et al., 2018). The model explains why humans appear to underestimate self-motion over short distances and overestimate self-motion over large distances. As we describe elsewhere in this article, uncertainty during the blackout from the present study may contribute to the elevated heading bias toward the preswitch heading in the midblackout condition.

### Flux in optic flow and naturalistic self-motion

The switch condition in the present study consists of optic flow that would be experienced during self-motion along a piecewise linear path. We acknowledge that a limited number of situations in the context of naturalistic self-motion and gaze patterns would give rise to such optic flow (Matthis, Muller, Bonnen, & Hayhoe, 2022). Nevertheless, humans may experience a similar shift when external forces, such as a strong gust of wind or water current, abruptly redirect the heading of a vehicle driven along a straight path. A similar phenomenon may arise when glancing a stationary object that causes a deflection in heading. Bias in heading perception toward the heading experienced before such a perturbation (preswitch heading) could be used to estimate the extent of the unexpected heading shift and support visual stability. In our experiment, subjects did not experience any transient rotation or inertial signals that likely arise in these naturalistic scenarios, however, which makes the correspondence with the experimental stimuli incomplete.

Naturalistic self-motion involves frequent saccadic eye movements, which induce sudden shifts in the singularity position within the optic flow field even when heading remains constant relative to the surrounding environment. For example, saccading to glance at the speedometer while driving down a straight road shifts the singularity upward in the optic flow field even though world-relative heading of the vehicle does not change. This raises the possibility that subjects may have perceived an invariant world-relative heading throughout Switch trials and a simulated saccade shifted the singularity on the eye of the simulated observer. In this scenario, the bias found in the present study would be toward the heading direction in retinal coordinates before the simulated saccade. Such a bias might support the stability of self-motion perceptual after the observer saccades back to the world-relative heading (e.g. saccade from the speedometer back to the road). Overall, however, we find the perception of a simulated saccade unlikely given that subjects maintained fixation during each trial and the visual system would need to tolerate a conflict between stationary position of the real eye and the shift in the optic flow.

It is also possible to experience a shift in the singularity position while moving along a curvilinear path. Curvilinear self-motion under naturalistic conditions often involves concomitant rotation of the body and gaze around the curved path that gives rise to a combination of translational and rotational optic flow (Warren, Mestre, Blackwell, & Morris, 1991; Royden, 1994; Raudies & Neumann, 2013). However, if the body and gaze remain parallel to a world-fixed direction during curvilinear self-motion, the observer experiences a radial optic flow field wherein the FoE drifts horizontally over time. Psychophysical judgments indicate that under such conditions humans perceive themselves moving along a straight rather than curved path (Li & Cheng, 2011b; Saunders, 2010). It seems implausible that subjects perceived themselves as moving along a curvilinear path in our study due to the lack of rotation in the optic flow and the fact that the FoE shifted once rather than drifted continuously.

### Serial dependence

The influence of previously viewed stimuli on human perceptual judgments occurs widely through what is known as serial dependence (Fischer & Whitney, 2014; Kiyonaga et al., 2017; Cicchini, Mikellidou, & Burr, 2018). The typical paradigm used to investigate this phenomenon analyzes the extent to which judgments exhibit bias based on the properties of stimuli in the past one or more trials (Kiyonaga et al., 2017). For example, Papadimitriou et al. (2017) trained monkeys to a saccade to the position of a target several seconds after it disappeared in a delayed-response task. The saccades exhibited bias toward the position of the target viewed on the previous trial and the amount of bias peaked when the target positions differed by 60${}^{\circ }$ across the successive trials.

Although the effect of the preswitch heading on heading judgments that is demonstrated in the present study could be considered “serial dependence”, we emphasize that we used a paradigm that is different from that commonly used in the serial dependence literature. In particular, we examined how a change in heading direction influences heading judgments within the *same* trial rather than *across* consecutive trials. We used this approach to address the stability of heading perception during a continuous period of simulated self-motion. Sun et al. (2020) used the “across-trial” approach and demonstrated that serial dependence arises in heading judgments from optic flow obtained across successive trials when each trial consists of simulated self-motion along a single heading direction. We argue that the paradigm of Sun et al. (2020) addresses different aspects of heading perception than those investigated here. Sun et al. (2020) examined the relationship between heading judgments obtained across two discrete episodes of simulated self-motion separated by a period in which subjects judged the heading on the previous trial. In contrast, the present study focuses on the temporal stability of heading perception during a continuous period of simulated self-motion. The two paradigms therefore create two different contexts (i.e. continuous vs discrete episodes of simulated self-motion) and unfold over different time scales. The latter is noteworthy because the time between the stimulus presentation and the subject response exerts a strong influence on serial dependence in judgments obtained between trials (Kiyonaga et al., 2017; Papadimitriou et al., 2017). Given these meaningful differences between the studies, our results cannot be taken to directly support or contradict those of Sun et al. (2020).

### Neural mechanisms

The correspondence between the CD model estimates and the human data in the present study raises the possibility that similar neural mechanisms could underlie heading perception when the direction of self-motion changes. Duffy and Wurtz (1997) found that the optic flow pattern tuning of individual MSTd neurons changed based the order of stimulus presentation and a population of neurons fired in darkness 100 to 350-ms after the optic flow ceased. Both findings would be expected if MSTd or a related area generated a temporally evolving heading signal. Interestingly, the 250-ms blackout period used in Experiment 2 falls within the 100- to 350-ms poststimulus window used by Duffy and Wurtz (1997), which supports the possibility that a population of neurons like the one identified by Duffy and Wurtz could be involved in heading perception during brief blackouts. It is noteworthy that the endpoints of the 100- to 350-ms window were chosen arbitrarily—it is possible that the MSTd neurons in the identified population could fire for extended blackouts. Future work should address human heading perception and the underlying neural mechanisms over longer blackout periods.

Paolini, Distler, Bremmer, Lappe, and Hoffmann (2000) examined the related issue of whether MSTd neurons exhibit temporal dependence in their responses as the optic flow smoothly morphs into a sequence of different patterns (e.g., radial expansion, circular clockwise rotation). Although they concluded that MSTd neurons do not exhibit temporal dependence, their stimulus paradigm did not match ours. Specifically, their “heading test” considered the neural response over the 833-ms period during which the FoE in radial optic flow fields smoothly drifted from one location to another. For example, in one case the FoE smoothly drifted from a −60${}^{\circ }$ leftward heading with a 60${}^{\circ }$ rightward heading over the 833-ms period, passing through all intermediary waypoints, such as a straight-ahead (0${}^{\circ }$) heading midway. To establish temporal dependence, the test required the firing rate at five specific times during the 833-ms morphing period to be larger than expected from the firing rate obtained when the initial and final headings were presented for the entire 833-ms duration. We found that the mean bias halved when the postswitch optic flow was presented for 500-ms compared with 250-ms (Figure 3b). By 833-ms, the bias may be largely eliminated. The smooth sweep of the FoE over an extended period of time in their paradigm might dampen the influence of the preswitch heading compared with the instantaneous shift between two headings in our study. This may explain the apparent discrepancy with Duffy and Wurtz (1997), who did not smoothly morph the optic flow.

Neural adaptation represents another possible mechanism, though it acts in diverse ways depending on the stimulus and brain area. For example, adaptation may exert either attractive and repulsive neural effects in different circumstances (Kohn & Movshon, 2004; Kohn, 2007; Dragoi et al., 2000). This makes it difficult to pinpoint the pattern of heading bias that the mechanism would predict, especially since the effects of adaptation have not been thoroughly studied in MSTd. It is noteworthy that neural adaptation could act synergistically with other mechanisms, such as those proposed by the CD model. Indeed, several versions of the CD model not simulated here contain adaptation at the level of the MT-MSTd synapse (Layton & Fajen, 2016a, 2020) and in MT speed tuning (Steinmetz et al., 2022). However, this additional mechanism was not needed to capture the human data in the present study.

## Supplementary Material

Supplement 1

Supplement 2

Supplement 3

Supplement 4

Supplement 5

## Supplementary materials

Supplementary Movie S1. Example of Switch Trial.

Supplementary Movie S2. Example of No-Switch Trial

Supplementary Movie S3. Example of Preblackout Trial.

Supplementary Movie S4. Example of Midblackout Trial.

Supplementary Movie S5. Example of Postblackout Trial.
